# Effects of Exogenous Potassium (K^+^) Application on the Antioxidant Enzymes Activities in Leaves of *Tamarix ramosissima* under NaCl Stress

**DOI:** 10.3390/genes13091507

**Published:** 2022-08-23

**Authors:** Yahui Chen, Shiyang Zhang, Shanfeng Du, Xiaomian Zhang, Guangyu Wang, Jiefan Huang, Jiang Jiang

**Affiliations:** 1 Collaborative Innovation Center of Sustainable Forestry in Southern China of Jiangsu Province, Nanjing Forestry University, Nanjing 210037, China; 2 Department of Forest Resources Management, Faculty of Science, University of British Columbia, Vancouver, BC V6T 1Z4, Canada; 3 Zhejiang Academy of Forestry, Hangzhou 310023, China

**Keywords:** NaCl stress, *Tamarix ramosissima*, exogenous potassium (K^+^), mitigate, NaCl poison

## Abstract

Saline soil is a worldwide distributed resource that seriously harms plants’ growth and development. NaCl is the most widely distributed salt in saline soil. As a typical representative of halophytes, *Tamarix ramosissima* Lcdcb (*T. ramosissima*) is commonly grown in salinized soil, and halophytes have different abilities to retain more K^+^ under salt stress conditions. Halophytes can adapt to different salt environments by improving the scavenging activity of reactive oxygen species (ROS) by absorbing and transporting potassium (K^+^). In this study, electron microscope observation, hydrogen peroxide (H_2_O_2_) and malondialdehyde (MDA) contents determination, primary antioxidant enzyme activity determination and transcriptome sequencing analysis were carried out on the leaves of *T. ramosissima* under NaCl stress at 0 h, 48 h and 168 h. The results showed that H_2_O_2_ and MDA contents increased in the 200 mM NaCl + 10 mM KCl and 200 mM NaCl groups, but the content increased the most in the 200 mM NaCl group at 168 h. In addition, the leaves of *T. ramosissima* in the 200 mM NaCl + 10 mM KCl group had the most salt secretion, and its superoxide dismutase (SOD), peroxidase (POD) and catalase (CAT) activities were all higher than those of the 200 mM NaCl group and significantly higher than those of the control group. According to the results of transcriptome sequencing, it was found that the expression of 39 genes related to antioxidant enzyme activity changed significantly at the transcriptional level. Among them, 15 genes related to antioxidant enzyme activities were upregulated, and 24 genes related to antioxidant enzyme activities were downregulated in the leaves of *T. ramosissima* when exogenous potassium (K^+^) was applied under NaCl stress for 48 h; when exogenous potassium (K^+^) was used for 168 h under NaCl stress, 21 antioxidant enzyme activity-related genes were upregulated, and 18 antioxidant enzyme activity-related genes were downregulated in *T. ramosissima* leaves. Based on the changes of expression levels at different treatment times, 10 key candidates differentially expressed genes (DEGs) (*Unigene0050462*, *Unigene0014843*, *Unigene0046159*, *Unigene0046160*, *Unigene0008032*, *Unigene0048033*, *Unigene0004890*, *Unigene0015109*, *Unigene0020552* and *Unigene0048538*) for antioxidant enzyme activities were further screened. They played an important role in applying exogenous potassium (K^+^) for 48 h and 168 h to the leaves of *T. ramosissima* in response to NaCl stress. Their expression levels were dominated by upregulation, which enhanced the activity of antioxidant enzymes, and helped *T. ramosissima* mitigate NaCl poison and resist NaCl stress. Particularly, *Unigene0048538* in glutathione S-transferase (GST) activity had the largest log_2_ fold-change in the comparison groups of 200 mM NaCl-48 h vs. 200 mM NaCl + 10 mM KCl-48 h and 200 mM NaCl-168 h vs. 200 mM NaCl + 10 mM KCl-168 h. Its expression level was upregulated and played an important role in NaCl toxicity. At the same time, the results of the phylogenetic tree analysis showed that *Unigene0048538* had the closest genetic distance to *Prunus persica* in the evolutionary relationship. In summary, with the increase of exogenous potassium (K^+^) application time under NaCl stress, *T. ramosissima* can resist high NaCl stress by enhancing antioxidant enzymes’ activity and maintaining the growth of *T. ramosissima.* Still, it is not enough to completely eliminate NaCl poison. This study provides a theoretical basis for the molecular mechanism of salt tolerance and K^+^ mitigation of NaCl poison by the representative halophyte *T. ramosissima* in response to NaCl stress.

## 1. Introduction

Soil salinization is one of the most important abiotic factors affecting plant growth, development and yield [1]. High concentrations of salt ions in salinized soils can cause a decrease in soil water potential and cause water stress to plants. Simultaneously, saline soils can produce ion stress, disrupt cell ion balance and interfere with ion metabolism. The saline soil environment can lead to a large accumulation of reactive oxygen species (ROS) in plants, damaging or even killing cells. Plants growing in salinized soils often suffer from nutrient stress due to a lack of potassium (K^+^), resulting in abnormal plant growth and development.

NaCl is one of the most widely distributed salts in saline soil, and it can negatively affect various physiology and biochemistry in plant growth and development stages. Munns [2] believed that there were three main reasons for the influence of salt on plant growth and development: Firstly, the low water potential in saline soil causes the water potential of plant leaves to drop, resulting in a decrease in stomatal conductance, which is the fundamental reason why salt affects various physiological and biochemical processes. Secondly, salt damage reduces photosynthesis rate and assimilation and energy supply, thereby limiting the growth and development of plants. Thirdly, salt damage affects some specific enzymes or metabolic processes, and the ability of plant protoplasts to retain potassium (K^+^) is considered an important sign of plant salt resistance [3]. Potassium (K^+^) is the plants’ second most abundant mineral element, accounting for 2–10% of a plant’s dry weight [4]. Studies have shown that among various macronutrients, K^+^ plays an essential role in the survival of plants under salt stress [5]. A well-balanced K^+^/Na^+^ ratio is necessary for properly regulating stomatal function, the activation of enzymes, protein synthesis, cellular osmoregulation, oxidant metabolism, photosynthesis and the maintenance of plant growth, which are essential [6]. Moreover, plants maintain a higher concentration of potassium (K^+^) in the cytoplasm, which promotes plant growth and improves plant salt tolerance. In saline soil, a high concentration of Na^+^ competes with plants to absorb potassium (K^+^), leading to potassium (K^+^) leakage through outward rectifying potassium (K^+^) channels, reducing the K^+^/Na^+^ ratio, causing Na^+^ poisoning in plants, resulting in biochemical metabolism disorders in plants, thereby affecting normal growth and development, resulting in plant growth inhibition or even death eventually [7,8]. It has been reported that the accumulation of Na^+^ in the wheat root under NaCl stress is reduced, and the leaves have a lower Na^+^/K^+^ ratio, which is considered an important feature of plant tolerance to salt stress. [9]. Therefore, increasing potassium (K^+^) uptake and reducing Na^+^ accumulation can better help plants resist salt stress [1,7].

Salt stress leads to the production of reactive oxygen species (ROS), such as O_2_^−^, OH^−^, O_2_^1^, etc., and hydrogen peroxide (H_2_O_2_) produced under salt stress can damage mitochondria and chloroplasts by disturbing cell structure [10,11]. An excessive accumulation of ROS can cause the membrane lipid peroxidation of unsaturated fatty acids in the membrane system. Its reaction product malondialdehyde (MDA) binds to proteins on the cell membrane and inactivate it, leading to cell death in severe cases. Plants produce some antioxidant enzymes under salt stress, such as superoxide dismutase (SOD), peroxidase (POD) and catalase (CAT), to protect themselves from the damaging effects of ROS [11]. Studies have shown that plant tolerance to salt is positively related to antioxidant systems [6]. Hence, we can see that the enhancement of plant antioxidant capacity can improve plant salt tolerance. Furthermore, potassium plays an important role in photosynthesis, protein synthesis, the regulation of plant stomata and water utilization, ion balance control, enzyme activations, etc. [6,12]. ROS generated by salt stress typically leads to lipid peroxidation and induces potassium (K^+^) loss from cells by activating potassium (K^+^) efflux channels [13,14]. It has been reported that improving the nutritional status of potassium in plants can significantly reduce the production of reactive oxygen species [6,15].

*Tamarix ramosissima* Lcdcb (*T*. *ramosissima*) is a halophyte that excretes salts. Its salt glands secrete salt directly to the leaf surface. Its salt glands are mainly distributed on stems and leaves, which can effectively discharge excess salt in cells [16]. During its long-term survival and evolution, *T*. *ramosissima* has been highly tolerant to various abiotic stresses such as salt, drought and high temperature. This suggests that it has developed efficient abiotic stress tolerance systems to adapt to adverse environments [17]. Salt glands are an important morphological feature of *T*. *ramosissima* to adapt to the saline–alkali environment and one of its main salt secretion mechanisms [18]. In addition, different halophytes have different abilities to retain more potassium (K^+^) under salt stress conditions [19]. Previous studies have found that NaCl stress below 100 mM can promote the growth of *T*. *ramosissima*, while NaCl stress above 200 mM inhibits the growth of *T*. *ramosissima* [20]. Additionally, the addition of exogenous 10 mM KCl can effectively alleviate the growth toxicity of *Alternanthera philoxeroides* (Mart. Griseb) under drought stress, increasing the potassium (K^+^) enrichment level in plants [21]. It was found that overexpression of *ApKUP4* in *Arabidopsis thaliana* significantly enhanced the K^+^ enrichment level and ROS scavenging ability of transgenic plants under NaCl stress conditions, thereby improving the tolerance of transgenic plants to NaCl stress [22]. In this study, the physiological and transcriptional data of *T*. *ramosissima* were combined to reveal the changes in salt secretion and antioxidant enzyme activities in the leaves of *T*. *ramosissima* in response to NaCl stress by exogenous potassium (K^+^) application and to explore critical genes and their potential regulatory networks and provide a scientific theoretical basis and the genetic resources for studying the damage-relieving effect of exogenous potassium (K^+^) on *Tamarix* plants under NaCl stress.

## 2. Materials and Methods

### 2.1. Plant Materials

The experiment was completed at the key laboratory, School of Forestry, Nanjing Forestry University from October 2019 to May 2021, and *T*. *ramosissima* seedlings were provided by Dongying Experimental Base of Shandong Academy of Forestry. We selected 5-month-old *T*. *ramosissima* seedlings with similar growth and transferred them to a 24-hole hydroponic box (40 cm × 30 cm × 16 cm) filled with 1/2 Hoagland’s nutrient solution. The culture medium was replaced every 3 days and placed in a greenhouse with a temperature of 26 ± 2 °C (day) and maintained at a relative humidity of 40% to 55%.

### 2.2. NaCl Treatment and NaCl + KCl Treatment

The control group and treatment groups were set in each experiment, with 8 strains in each group, and the experiment was repeated three times in total. They were cultured with 1/2 Hoagland’s nutrient solution as the control group. We added 1/2 Hoagland’s nutrient solution of 200 mM NaCl and 1/2 Hoagland’s nutrient solution of 200 mM NaCl + 10 mM KCl as the treatment group, changing the culture medium every 3 days. Then, the *T*. *ramosissima* root samples were collected at 0 h, 48 h and 168 h after treatment, immediately placed in liquid nitrogen for treatment and then transferred to a −80 °C refrigerator for storage for later use.

### 2.3. Analysis of Leaf Salt Secretion under Different Treatments of T. ramosissima

We collected leaf materials from the control and treatment groups at 0 h, 48 h and 168 h, respectively, and used a JSZ6S stereo microscope (Jiangnan, Nanjing, China) to observe the distribution of salt secretion on the leaf surface for analysis.

### 2.4. Determination and Analysis of Antioxidant Enzyme Activity

The leaves of 8 plants were randomly selected at 48 h and 168 h after treatment, and each treatment had 3 biological replicates to measure H_2_O_2_ and MDA contents, SOD, POD and CAT activities [23]. The antioxidant enzyme activity was studied via the physiological mechanism of alleviation of *T. ramosissima*, by exogenous potassium (K^+^) application under NaCl stress.

### 2.5. Transcriptome Sequencing and Differentially Expressed Gene Screening

The leaf samples of *T*. *ramosissima* were treated with liquid nitrogen and sent to Guangzhou GENE Denovo Company for 3-generation high-throughput transcriptome sequencing. Referring to Chen’s transcriptome sequencing method [23], the obtained raw sequencing data were submitted to the National Center for Biotechnology Information (NCBI) Short Reads Archive (SRA) database, and the SRP number was SRP356215. The reads count data obtained by sequencing were analyzed using DESeq2 [24] to obtain the final correct FDR value (FDR value is the *p*-value after BH correction), and a corrected *p* < 0.05 was considered to be significantly enriched. Based on the differential analysis results, we screened out genes as significantly DEGs according to the criteria of FDR < 0.05 and |log2FC| > 1. Finally, the DEGs were used for Gene Ontology (GO) and Kyoto Encyclopedia of Genes and Genomes (KEGG) enrichment analyses using the GO database and release 93.0, respectively [25,26].

### 2.6. Validation of Real-Time Quantitative Reverse Transcription PCR (qRT-PCR)

Ten DEGs (*Unigene0104732, Unigene0083695, Unigene0069097, Unigene0024962, Unigene0007135, Unigene0088781, Unigene0028215, Unigene0082586, Unigene0003066* and *Unigene0090596*) were randomly selected for quantitative reverse transcription PCR (qRT-PCR) to verify the accuracy of RNA-seq results. We extracted total RNA from leaf material using Omega kit (Beinuo Bio, Shanghai, China) from Omega Bio-Tek. The primers were reverse-transcribed into 1-strand cDNA using PrimerScript^TM^ RT Master Mix (Perfect Real Time) kit from TaKaRa Company (Bao Bio, Dalian, China) of Bao Bioengineering Co., Ltd. as a template. DEGs-specific expression primers were designed (Supplemental Table S1), and the PowerUp^TM^ SYBR Green Master mix reagent from Thermo Fisher Scientific Co., Ltd. (Thermo Fisher, Shanghai, China) was used for the samples, the qRT-PCR detection was performed on the ABI ViiA™ 7 real-time PCR system (ABI, Carlsbad, CA, USA) of Applied Biosystems, and each candidate genes was subjected to 4 technical replicates for a total of 3 biological replicates. Using actin as the internal reference gene, the relative expression level was calculated by the 2^−ΔΔCt^ method [23].

## 3. Results

### 3.1. Analysis of Salt Secretion of T. ramosissima Leaves under Different Treatments

**Figure 1:**
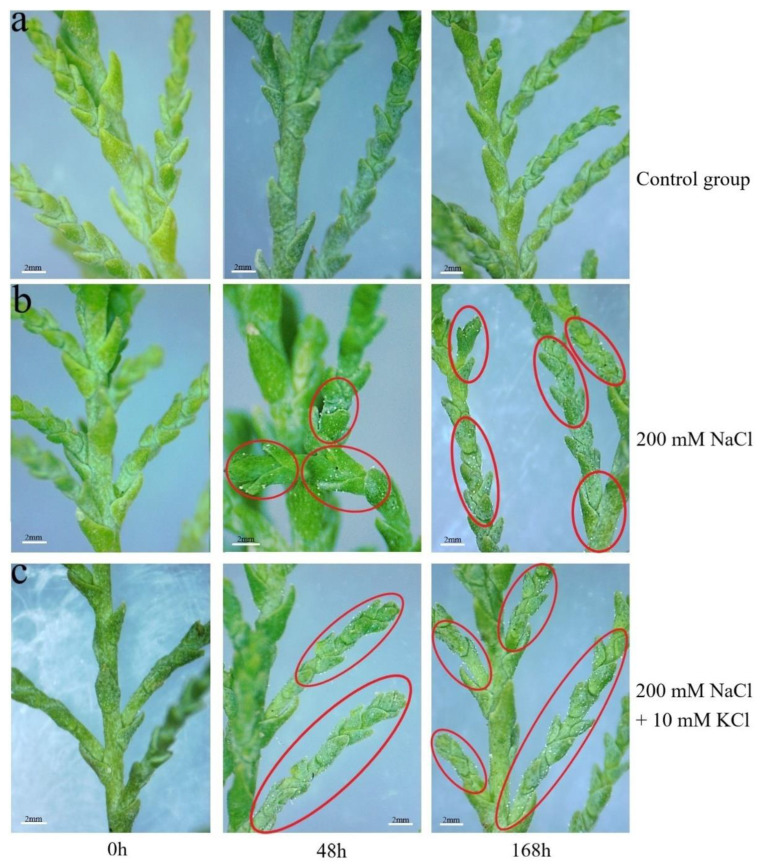
Changes in salt secretion in leaves of *T*. *ramosissima* under different treatments. Changes of salt secretion in leaves of *T*. *ramosissima* at 0 h, 48 h and 168 h under different treatments. Note: (**a**) represents the control group, (**b**) represents 200 mM NaCl, and (**c**) represents 200 mM NaCl + 10 mM KCl. The red circled parts indicate the salt secretion distribution of *T*. *ramosissima* leaves under different treatments. (**a**,**b**) refer to the data of Chen et al. (https://doi.org/10.1371/journal.pone.0265653).

There was no leaf salt secretion phenomenon in the control group at 48 h and 168 h, while the 200 mM NaCl group and 200 mM NaCl + 10 mM KCl group appeared to have a salt secretion phenomenon in leaves at 48 h and 168 h. With the increase of time, the leaves in the 200 mM NaCl + 10 mM KCl group had more salt secretion than the 200 mM NaCl group. Among them, the 200 mM NaCl group and the 200 mM NaCl + 10 mM KCl group began to show a leaf salt secretion phenomenon at 48 h; at 168 h, leaves in the 200 mM NaCl + 10 mM KCl group had the most salt secretion (Figure 1).

### 3.2. Analysis of H_2_O_2_ and MDA Content in T. ramosissima Leaves under Different Treatments

**Figure 2:**
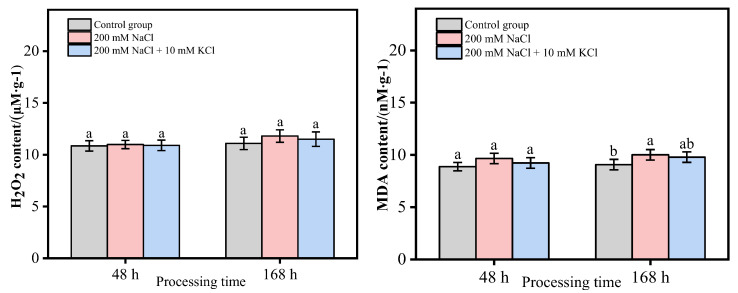
Changes of H_2_O_2_ and MDA content in leaves of *T. ramosissima* under different treatments. Changes of H_2_O_2_ and MDA content in the leaves of *T. ramosissima* at 48 h and 168 h in the control group and different treatment groups. Note: different letters indicate the significance of differences among treatments at the same time, *p* < 0.05.

In this study (Figure 2), within 168 h, the H_2_O_2_ content did not change significantly in the control group; the H_2_O_2_ content in the 200 mM NaCl and 200 mM NaCl + 10 mM KCl groups showed an increasing trend over time. At 48 h and 168 h, there were no significant changes in the 200 mM NaCl and 200 mM NaCl + 10 mM KCl groups compared with the control group, but the H_2_O_2_ content of the 200 mM NaCl group was higher than that of the 200 mM NaCl + 10 mM KCl group. Within 168 h, the MDA content in the control group showed a slowly increasing trend, but there was no significant change. The MDA content in the 200 mM NaCl and 200 mM NaCl + 10 mM KCl groups showed an increasing trend over time. At 48 h, the MDA content of the 200 mM NaCl and 200 mM NaCl + 10 mM KCl groups had no significant changes compared with that of the control group. At 168 h, the MDA content of the 200 mM NaCl group was higher than that of the control group, and there was a significant difference. Still, the MDA content of the 200 mM NaCl + 10 mM KCl group was slightly increased compared to the control group, but there was no significant difference.

In conclusion, the 200 mM NaCl group had the highest H_2_O_2_ content and MDA content within 168 h.

### 3.3. Antioxidant Enzyme Activity Analysis of T. ramosissima Leaves under Different Treatments

**Figure 3:**
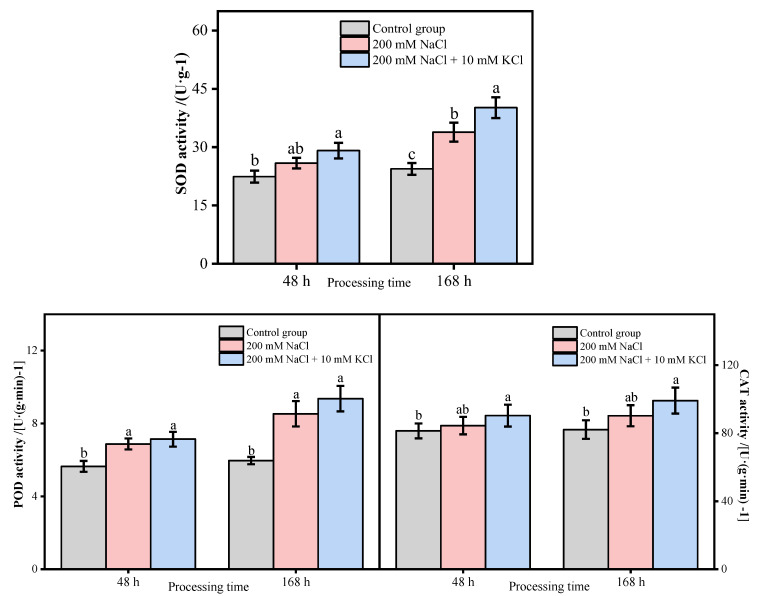
Changes of SOD, POD and CAT activities in leaves of *T*. *ramosissima* under different treatments. Changes of SOD, POD and CAT activities in leaves of *T. ramosissima* at 48 h and 168 h under different treatments. Note: different letters indicate the significance of differences among treatments at the same time, *p* < 0.05. There were 3 biological replicates applied for all determination.

The SOD activity in the control group did not change significantly within 168 h; the SOD activity in the 200 mM NaCl and 200 mM NaCl + 10 mM KCl groups showed an increasing trend with time. Among them, the SOD activity of the 200 mM NaCl + 10 mM KCl group increased the most. The SOD activity of the groups increased more than that of the control group at 168 h, with a significant difference. The 200 mM NaCl and 200 mM NaCl + 10 mM KCl groups also significantly differed in SOD activity at 168 h. The POD activity in the control group did not change significantly within 168 h. The POD activity in the 200 mM NaCl and 200 mM NaCl + 10 mM KCl groups showed a slowly increasing trend within 168 h. Among them, the POD activity increased the most in the 200 mM NaCl + 10 mM KCl group. Significantly, at 48 h and 168 h, the POD activity of the 200 mM NaCl and 200 mM NaCl + 10 mM KCl groups significantly increased compared with that of the control group. The CAT activity in the control group did not change significantly within 168 h; the CAT activity showed an upward trend in the 200 mM NaCl and 200 mM NaCl + 10 mM KCl groups over time. Among them, the 200 mM NaCl + 10 mM KCl group increased the most in CAT activity. At 48 h and 168 h, the CAT activity of the 200 mM NaCl group was higher than that of the control group, but there was no significant difference. However, the CAT activity in the 200 mM NaCl + 10 mM KCl group was higher than that in the control group, and there was a significant difference. Notably, within 168 h, the activities of the three antioxidant enzymes of the 200 mM NaCl + 10 mM KCl group were higher than those in the 200 mM NaCl group and were significantly higher than those in the control group (Figure 3).

### 3.4. KEGG Pathway Analysis of DEGs

**Figure 4:**
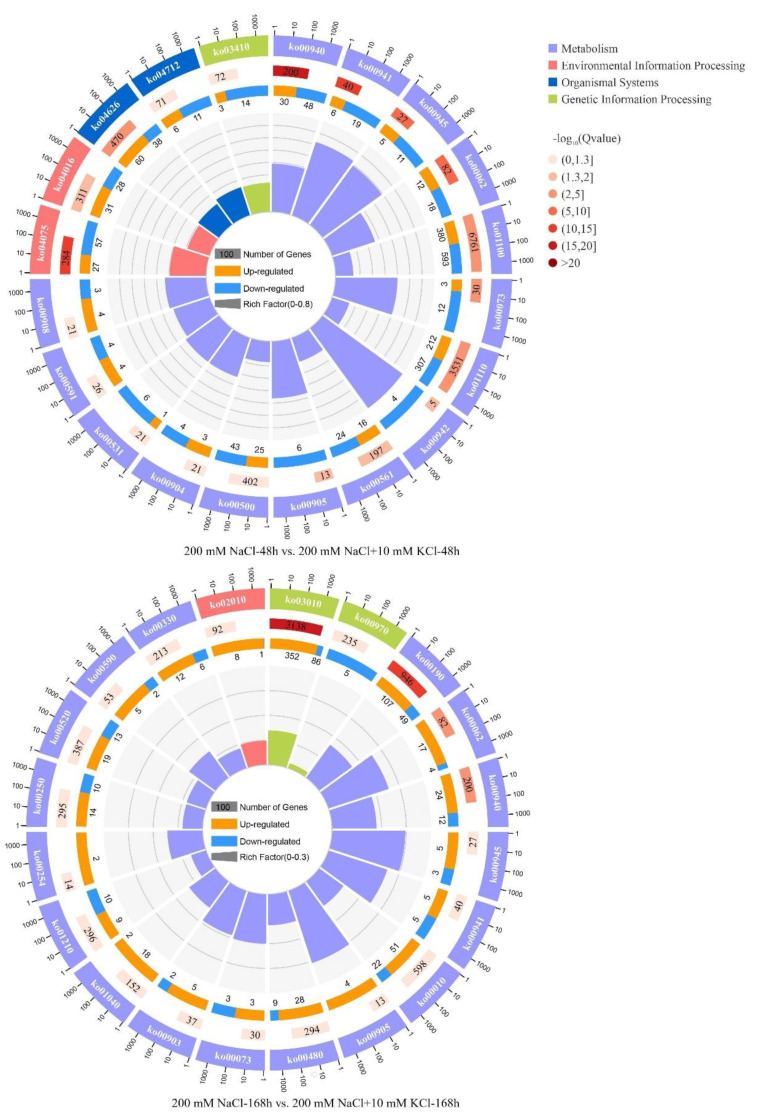
Top 20 KEGG pathways analysis in the transcriptome. First and outer circle: the top 20 KEGG pathways are enriched, outside the circle is the scale of the number of genes. Different colors represent different ontologies. Second circle: the number of the KEGG pathway in the background gene and the Q value. Moreover, the darker the color, the smaller the Q value. The longer the bars, the more genes it contains. The dark color represents the proportion of upregulated genes, and the light color represents the proportion of downregulated genes. The specific value is displayed below. Fourth and inner circle: the ratio of each KEGG pathway rich factor value (the number of differential genes in this KEGG pathway divided by all numbers); background grid lines, each grid represents 0.1.

Based on the KEGG pathway analysis for the 200 mM NaCl-48 h vs. 200 mM NaCl + 10 mM KCl-48 h and 200 mM NaCl-168 h vs. 200 mM NaCl + 10 mM KCl-168 h comparison groups, it is more intuitive to reflect the changes in the expression levels of related DEGs involved in the KEGG Pathway at 48 h and 168 h of the exogenous potassium (K^+^) application in *T*. *ramosissima* leaves under NaCl stress (Table 1). In the comparison groups, 200 mM NaCl-48 h vs. 200 mM NaCl + 10 mM KCl-48 h and 200 mM NaCl-168 h vs. 200 mM NaCl + 10 mM KCl-168 h, eight KEGG pathways were found to be the same, including Phenylpropanoid biosynthesis (ko00940), Plant hormone signal transduction (ko04075), Flavonoid biosynthesis (ko00941), Stilbenoid, diarylheptanoid and gingerol biosynthesis (ko00945), Fatty acid elongation (ko00062), Cutin, suberine and wax biosynthesis (ko00073), Brassinosteroid biosynthesis (ko00905) and Diterpenoid biosynthesis (ko00904), respectively. Among them, Phenylpropanoid biosynthesis (ko00940), Flavonoid biosynthesis (ko00941), Stilbenoid, diarylheptanoid and gingerol biosynthesis (ko00945), Fatty acid elongation (ko00062) and Brassinosteroid biosynthesis (ko00905) were significantly enriched by DEGs. In the 200 mM NaCl-48 h vs. 200 mM NaCl + 10 mM KCl-48 h comparison group, 2078 DEGs were changed in the top 20 KEGG pathways, 828 DEGs were upregulated and 1250 DEGs were downregulated; there were 15 KEGG pathway classifications to Metabolism, 2 of them belonged to Environmental Information Processing, 2 of them belonged to Organic Systems, and 1 of them belonged to Genetic Information Processing. In the 200 mM NaCl-168 h vs. 200 mM NaCl + 10 mM KCl-168 h comparison group, 974 DEGs were changed, 727 DEGs were upregulated, and 247 DEGs were downregulated in the top 20 KEGG Pathways. There were 18 KEGG pathways classified as Metabolism, 1 of them belonged to Environmental Information Processing, and 1 of them belonged to Genetic Information Processing (Figure 4).

To summarize, *T*. *ramosissima* leaves were exposed to exogenous potassium for 48 h and 168 h under NaCl stress. With the increased time, a large number of DEGs were involved in the Metabolism-related KEGG pathway, and the number of upregulated DEGs significantly increased. This indicated that the upregulated genes in the leaves of *T*. *ramosissima* were mainly regulated, and related DEGs were involved in the KEGG pathway and resisted NaCl injury.

### 3.5. Analysis of DEGs in Leaves of T. ramosissima by Exogenous Potassium (K^+^) Application under NaCl Stress

**Figure 5:**
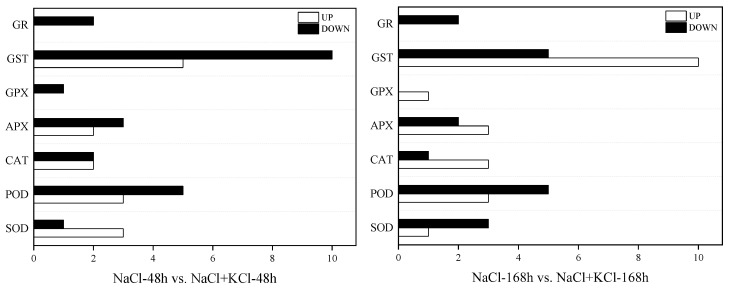
The number of gene expression changes of antioxidant enzymes in leaves of *T*. *ramosissima* under NaCl stress with exogenous potassium (K^+^) application. Changes in the expression levels of DEGs with 7 types of antioxidant enzymes in the leaves of *T*. *ramosissima* under NaCl stress for 48 h and 168 h. Note: NaCl means 200 mM NaCl; NaCl + KCl means 200 mM NaCl + 10 mM KCl. GR: glutathione reductase; GST: glutathione S-transferases; GPX: glutathione peroxidase; APX: ascorbate peroxidase; CAT: catalases; POD: peroxidase; SOD: superoxide dismutase.

According to the application of exogenous potassium (K^+^) in 48 h and 168 h of NaCl stress in *T*. *ramosissima* leaves, the results showed the expression of 39 ROS-scavenging-related DEGs in the leaves changed (Table 2). In the 200 mM NaCl-48 h vs. 200 mM NaCl + 10 mM + KCl-48 h comparison group, there were 15 upregulated genes and 24 downregulated genes. Among them, the highest number of upregulated genes was for glutathione S-transferase (GST) (5), followed by SOD (3), POD (3), CAT (2) and APX (2); the highest number of downregulated genes was for GST (10), followed by POD (5), ascorbate peroxidase (APX) (3), CAT (2), SOD (1), glutathione peroxidase (GPX) (1) and glutathione reductase (GR) (2) (Figure 5). In the 200 mM NaCl-168 h vs. 200 mM NaCl + 10 mM KCl-168 h comparison group, there were 21 upregulated genes and 18 downregulated genes. Among them, the highest number of upregulated genes was for GST (10), followed by POD (3), CAT (3), APX (3), SOD (1) and GPX (1); POD (5) had the largest number of downregulated genes along with GST (5), followed by SOD (3), APX (2), GR (2) and CAT (1) (Figure 5). In addition, the expression levels of *POD* (*Unigene0013825* and *Unigene0029752*), *CAT* (*Unigene0087092*), *APX* (*Unigene0008513*), *GPX* (*Unigene0035407*) and *GST* (*Unigene0001041*, *Unigene0012650*, *Unigene0064942*, *Unigene0069060*, *Unigene0082147* and *Unigene0098941*) were downregulated at 48 h under NaCl stress application of exogenous potassium (K^+^) but upregulated at 168 h under NaCl stress application of exogenous potassium (K^+^). However, *SOD* (*Unigene0050462*), *POD* (*Unigene0014843*), *CAT* (*Unigene0046159* and *Unigene0046160*), *APX* (*Unigene0008032* and *Unigene0048033*) and *GST* (*Unigene0004890, Unigene0015109, Unigene0020552* and *Unigene0048538*) were upregulated during 48 h and 168 h of exogenous potassium (K^+^) application under NaCl stress. In particular, *Unigene0048538* had the largest log_2_ fold-change in the 200 mM NaCl-48 h vs. 200 mM NaCl + 10 mM KCl-48 h and 200 mM NaCl-168 h vs. 200 mM NaCl + 10 mM KCl-168 h comparison groups. The results showed that the leaves of *T*. *ramosissima* were exposed to exogenous potassium (K^+^) for 48 h and 168 h under NaCl stress. With the increase of time, its antioxidant mechanism was affected by exogenous potassium (K^+^) via upregulating the expression of antioxidant genes to initiate corresponding physiological responses to alleviate the damage caused by NaCl (Figure S2).

### 3.6. Phylogenetic Tree Analysis of Key Candidate Genes in Antioxidative Enzyme Activity

**Figure 6:**
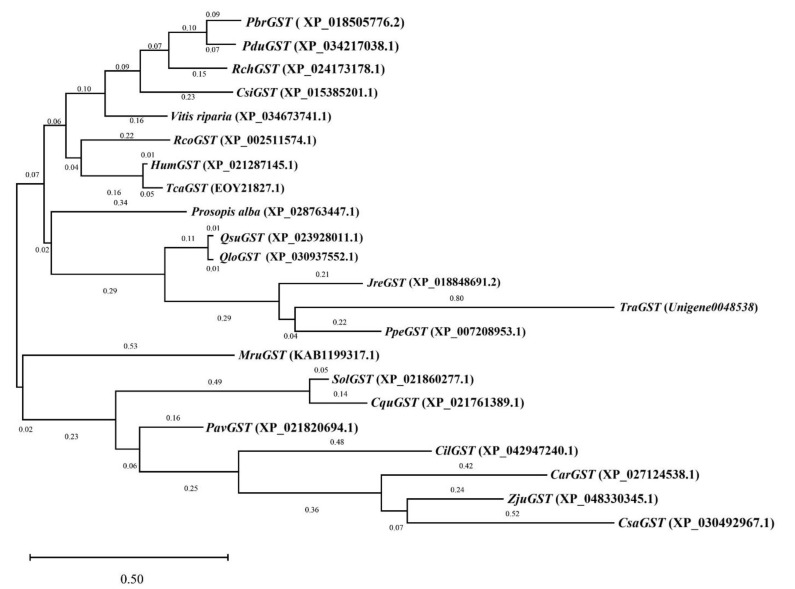
Phylogenetic tree analysis of *T*. *ramosissima* GST and other species GST. Phylogenetic tree analysis of Unigene0048538 protein amino acid sequence and protein amino acid sequence of other 21 species of *T*. *ramosissima.*

Section 3.5 showed that *Unigene0048538* had the largest log_2_ fold-change in the 200 mM NaCl-48 h vs. 200 mM NaCl + 10 mM KCl-48 h and 200 mM NaCl-168 h vs. 200 mM NaCl + 10 mM KCl-168 h comparison groups in GST activity. Its expression level was upregulated and played an important role in NaCl toxicity (Supplemental Figure S1). Therefore, using BLAST, the protein amino acid sequence of *Unigene0048538* was selected for alignment in the National Center for Biotechnology Information (NCBI). In this study, 21 homologous gene species were selected (Table 3), and using MEGA software (MEGA Software, Pennsylvania, USA), the phylogenetic tree was constructed by combining the amino acid sequence of *Unigene0048538* protein of *T*. *ramosissima* with the amino acid sequences of these 21 homologous gene species (Figure 6). The results showed that *Unigene0048538* had the closest genetic distance to *Prunus persica* in the evolutionary relationship.

### 3.7. Real-Time Quantitative Reverse Transcription PCR (qRT-PCR) Validation

This study selected 10 DEGs (Supplemental Table S2) for qRT-PCR verification. The results showed that these qRT-PCR verification results were completely consistent with the expression trend of the transcriptome sequencing analysis results (Supplemental Figure S1). The results showed that the transcriptome data obtained in this study were accurate and reliable and could provide gene resources and transcriptional level basis for studying the mechanism of exogenous potassium (K^+^) application under NaCl stress in *T*. *ramosissima* to alleviate salt toxicity and salt tolerance.

## 4. Discussion

*Tamarix* plants are halophytes that excrete salt, and the salt gland is one of the important morphological characteristics of the *T. ramosissima* ability to adapt to the saline-alkali environment [27]. Its salt glands secrete salt directly to the leaf surface, which can effectively remove excess salt in cells and reduce the damage of ion poisoning to plants [16]. This study’s electron microscopy analysis result showed that leaf salt secretion increased with the prolongation of NaCl stress time. Particularly, the addition of exogenous potassium (K^+^) increased the rate of salt secretion of *T*. *ramosissima*, suggesting that *T*. *ramosissima* could alleviate and respond to the toxicity of salt stress through the phenomenon of salt secretion. In particular, adding exogenous potassium (K^+^) could speed up the mitigation and adaptation of *T*. *ramosissima* to prolonged high-salt stress. *Tamarix* plants have evolved a series of complex mechanisms to resist salt stress during the long-term adaptation process to the environment. Plants’ salt stress responses are complex processes involving and regulated by multiple genes at the molecular level. There are few reports on the molecular mechanism of applying exogenous potassium (K^+^) in *T*. *ramosissima* under NaCl stress. Studies have shown that using potassium (K^+^) fertilizer under salt stress conditions can improve plant growth [6]. Therefore, the transcriptome sequencing analysis of the leaves of *T*. *ramosissima* under NaCl stress with an exogenous potassium (K^+^) application can help to reveal the response of *Tamarix* plants to the salt stress environment and the mechanism of potassium (K^+^) alleviating salt toxicity.

Plants are usually subjected to various adverse factors during the growth process, which produces a large amount of ROS in the plant. The main types of ROS are H_2_O_2_, OH^−^, O^2−^, etc. Excessive accumulation of ROS can lead to oxidative stress, damage biological macromolecules, biofilms and other structures, and even lead to cell death in severe cases. The increase in the content of various antioxidant enzymes is an important physiological mechanism for plants to adapt to the salt environment. Plants scavenge reactive oxygen species through changes in the activity of the protective enzyme system, prevent membrane lipid peroxidation and inhibit the accumulation of MDA content in the membrane. Hence, plants of the salt-tolerant genotype detoxify by increasing the antioxidant defense system [28,29,30]. In this study, the H_2_O_2_ and MDA contents increased in both 200 mM NaCl and 200 mM NaCl + 10 mM KCl groups within 168 h, but the 200 mM NaCl group had the most H_2_O_2_ and MDA contents. In addition, SOD, POD and CAT activities all increased in the 200 mM NaCl and 200 mM NaCl + 10 mM KCl groups. In particular, the activities of these three antioxidant enzymes in the (200 mM NaCl + 10 mM KCl)-treated group were higher than those in the (200 mM NaCl)-treated group and were significantly higher than those in the control group. In order to explore the response of DEGs related to the antioxidant system in *T*. *ramosissima* to NaCl stress and the changes in expression levels of adding exogenous potassium (K^+^) to mitigate NaCl poison, in this study, the leaves of *T*. *ramosissima* were exposed to exogenous potassium (K^+^) under NaCl stress, and the expressions of 39 ROS scavenging-related DEGs were significantly changed. Among them, the application of exogenous potassium (K^+^) for 48 h under NaCl stress of *T*. *ramosissima* resulted in the upregulation of 15 genes and the downregulation of 24 genes; the application of exogenous potassium (K^+^) for 168 h under NaCl stress resulted in the upregulation of 21 genes and the downregulation of 18 genes. These DEGs have regulatory effects on the physiological mechanism of salt tolerance and alleviation of NaCl poison in *T*. *ramosissima*.

Maintaining the integrity of the membrane system is the key to a plant’s salt resistance. In the long-term evolution process, plants have correspondingly formed two major reactive-oxygen-species-scavenging systems, enzymatic systems and nonenzymatic systems. SOD, CAT and POD are important enzymes for ROS metabolism in plants, which can slow down or resist the damage of adversity to plants to a certain extent [31]. Under salt stress, plants can use antioxidant enzymes such as SOD, POD and CAT to reduce or remove ROS damage [32]. In this study, three DEGs (*Unigene0033269, Unigene0050462* and *Unigene0082550*) in SOD activity, three DEGs (*Unigene0014843, Unigene0049353* and *Unigene0094375*) in POD activity and two DEGs (*Unigene0046159* and *Unigene0046160*) in CAT activity were upregulated in the 200 mM NaCl-48 h vs. 200 mM NaCl + 10 mM KCl-48 h comparison group; one DEG (*Unigene0050462*) in SOD activity, three DEGs (*Unigene0013825, Unigene0014843* and *Unigene0029752*) in POD activity and three DEGs (*Unigene0046159, Unigene0046160* and *Unigene0087092*) in CAT activity were upregulated in the 200 mM NaCl-168 h vs. 200 mM NaCl + 10 mM KCl-168 h comparison group. Among them, four DEGs, including *Unigene0050462* in SOD activity, *Unigene0014843* in POD activity and *Unigene0046159* and *Unigene0046160* in CAT activity were upregulated by exogenous potassium (K^+^) for 48 h and 168 h under NaCl stress. This suggests that these DEGs are affected by exogenous potassium (K^+^) and play a role in alleviating NaCl poison. As in previous studies, the upregulation of the expression levels of these three types of enzyme-related genes will promote the enhancement of enzyme activity, enhance the ability to scavenge ROS, and thus improve the resistance of plants to abiotic stress [33]. Ascorbate peroxidase (APX) and glutathione peroxidase (GPX) can protect cells by catalyzing H_2_O_2_ under plant stress and are the key enzymes for scavenging ROS. Studies have shown that APX family genes contribute to plant tolerance mechanisms during salt stress [34]. The overexpression of ascorbate peroxidase in tobacco chloroplasts enhances tolerance to salt stress and deficiency [35]. Moreover, potassium (K^+^) application can improve the scavenging system’s ability to scavenge ROS, especially the activities such as GPX [6,36]. *NtPHGPX* in tobacco can enhance salinity tolerance by scavenging ROS accumulation and redox balance in the body membrane [37]. It has also been reported that *LePHGPX* can inhibit salt, heat shock and Bax protein-induced cell death, thereby protecting plant tissues from various stresses [38]. In this study, two DEGs (*Unigene0008032* and *Unigene0048033*) in APX activity were upregulated in the 200 mM NaCl-48 h vs. 200 mM NaCl + 10 mM KCl-48 h comparison group; There were three DEGs in APX activity (*Unigene0008032*, *Unigene0008513* and *Unigene0048033*), and one DEG in GPX activity up-regulated in the 200 mM NaCl-168 h vs. 200 mM NaCl + 10 mM KCl-168 h comparison group (*Unigene0035407*). This indicated that both APX and GPX were involved in plant response to salt stress. With the increase of exogenous potassium (K^+^) time addition, more APX- and GPX-related DEGs resisted NaCl stress by upregulating their expression levels. In APX activity, *Unigene0008032* and *Unigene0048033* were upregulated under NaCl for 48 h and 168 h, indicating that these two DEGs played a role in resistance to salt stress in *T*. *ramosissima*. Glutathione S-transferase (GST) protects plants from different abiotic stresses and adversities, and its main functions are detoxification and antioxidant defense [39]. Studies have shown that the expression of related genes in GST activity is increased, the chloroplast antioxidant defense system is enhanced, and the salt tolerance ability of plants is improved [40]. GST-activity-related genes are sensitive to a high concentration of salt (≥200 mM) stress. Still, the related genes in GST activity are not sensitive to low and medium concentrations of salt (≤100 mM) stress [41], and the expression level of related genes in GST activity is upregulated under salt stress, which can improve the tolerance of plants under salt stress [42]. Five DEGs in GST activity in this study (*Unigene0004890*, *Unigene0015109*, *Unigene0020552*, *Unigene0048538* and *Unigene0069058*) were upregulated in the 200 mM NaCl-48 h vs. 200 mM NaCl + 10 mM KCl-48 h comparison group; ten DEGs in GST activity (*Unigene0001041*, *Unigene0004890*, *Unigene0012650*, *Unigene0015109*, *Unigene0020552*, *Unigene0048538*, *Unigene0064942*, *Unigene0069060*, *Unigene0082147* and *Unigene0098941*) were upregulated in 200 mM NaCl-168 h vs. 200 mM NaCl + 10 mM KCl level comparison group. In particular, four DEGs in GST activity including *Unigene0004890*, *Unigene0015109*, *Unigene0020552* and *Unigene0048538* showed upregulated expression levels under NaCl application of exogenous potassium (K^+^) for 48 h and 168 h. These results indicated that with the increase of exogenous potassium (K^+^) addition time under NaCl stress, a large number of genes in GST activity were activated, and the expression level was dominated by the upregulation, which resisted NaCl stress and maintained plant growth. Plant photosynthesis is reduced under high salt stress, and the reduction in photosynthetic pigment content exposes plants to excessive light energy, which accelerates the production of ROS and causes damage to cell membranes. The activities of CAT, APX and GR are insufficient to exceed the production of H_2_O_2_ [43]. It has been reported that the expression of the tea tree *CsGR2* gene is downregulated in response to salt stress [44], which is similar to the results of this study. In this study, *Unigene0075696* and *Unigene0098587* in GR activity were downregulated in the 200 mM NaCl-48 h vs. 200 mM NaCl + 10 mM KCl-48 h and 200 mM NaCl-168 h vs. 200 mM NaCl + 10 mM KCl-168 h comparison groups. It is inferred from this that adding exogenous potassium (K^+^) alleviated the NaCl poison to a certain extent under high NaCl stress but could not completely eliminate the NaCl poison. Therefore, the GR activity was decreased.

## 5. Conclusions

*T*. *ramosissima* leaves showed salt secretion in the 200 mM NaCl + 10 mM KCl and 200 mM NaCl groups, and the H_2_O_2_ and MDA contents and activities of SOD, POD and CAT increased. Among them, the H_2_O_2_ and MDA contents of the 200 mM NaCl group was the highest at 168 h. Notably, the leaves in the 200 mM NaCl + 10 mM KCl group had the most salt secretion, and the activities of SOD, POD and CAT were all higher than those in the 200 mM NaCl group, which were significantly higher than those in the control group. According to the transcriptome sequencing of *T*. *ramosissima* leaves in response to exogenous potassium (K^+^) application under NaCl stress, it was found that the expression of 39 DEGs related to antioxidant enzyme activities changed significantly at the transcriptional level. Fifteen DEGs related to antioxidant enzyme activities were upregulated, and twenty-four DEGs related to antioxidant enzyme activities were downregulated in the leaves of *T*. *ramosissima* under NaCl stress for 48 h. Twenty-one DEGs related to antioxidant enzyme activities were upregulated, and eighteen DEGs related to antioxidant enzyme activities were downregulated when exogenous potassium (K^+^) was applied to tamarind leaves under NaCl stress for 168 h. A further screening was conducted to obtain 10 key candidate DEGs (*Unigene0050462*, *Unigene0014843*, *Unigene0046159*, *Unigene004616*0, *Unigene0008032*, *Unigene0048033*, *Unigene0004890*, *Unigene0015109*, *Unigene0020552* and *Unigene0048538*) for antioxidant enzyme activities. In response to NaCl stress, they played an important role in 48 h and 168 h of exogenous potassium (K^+^) application in *T*. *ramosissima* leaves. They mainly upregulated the expression level, enhanced the activity of antioxidant enzymes and helped *T*. *ramosissima* mitigate NaCl poison. Particularly, *Unigene0048538* in GST activity had the largest log_2_ fold-change in the comparison groups of 200 mM NaCl-48 h vs. 200 mM NaCl + 10 mM KCl-48 h and 200 mM NaCl-168 h vs. 200 mM NaCl + 10 mM KCl-168 h. Its expression level was upregulated and played an important role in NaCl toxicity. At the same time, the results of the phylogenetic tree analysis showed that *Unigene0048538* had the closest genetic distance to *Prunus persica* in the evolutionary relationship.

In conclusion, with the increase of the exogenous potassium (K^+^) application time under NaCl stress, *T*. *ramosissima* can resist high NaCl stress by enhancing the activity of antioxidant enzymes to maintain the growth of *T*. *ramosissima*. Still, it is not enough to completely eliminate the toxicity of NaCl. This study provides a scientific basis for further research on the salt tolerance and K^+^ alleviation molecular mechanism of exogenous potassium (K^+^) in *T*. *ramosissima* under NaCl stress.

## Figures and Tables

**Table 1:** Annotation of DEGs in the top 20 KEGG pathways of *T*. *ramosissima* leaves under NaCl stress with exogenous potassium (K^+^).

ID	Gene Numbers	Class	KEGG Pathway	*p*-Value	Up	Down
**200 mM NaCl-48 h vs. 200 mM NaCl + 10 mM KCl-48 h**						
Phenylpropanoid biosynthesis	78	Metabolism	ko00940	0.000000	30	48
Plant hormone signal transduction	84	Environmental Information Processing	ko04075	0.000000 0.000000	27	57
Flavonoid biosynthesis	25	Metabolism	ko00941	0.000000	6	19
Stilbenoid, diarylheptanoid and gingerol biosynthesis	16	Metabolism	ko00945	0.000000	5	11
Fatty acid elongation	30	Metabolism	ko00062	0.000000	12	18
Plant–pathogen interaction	98	Organismal Systems	ko04626	0.000000	60	38
Metabolic pathways	973	Metabolism	ko01100	0.000000	380	593
Cutin, suberine and wax biosynthesis	15	Metabolism	ko00073	0.000000	3	12
Biosynthesis of secondary metabolites	519	Metabolism	ko01110	0.000193	212	307
Anthocyanin biosynthesis	4	Metabolism	ko00942	0.001236	0	4
MAPK signaling pathway—plant	59	Environmental Information Processing	ko04016	0.001374	31	28
Glycerolipid metabolism	40	Metabolism	ko00561	0.002193	16	24
Brassinosteroid biosynthesis	6	Metabolism	ko00905	0.003482	0	6
Circadian rhythm-plant	17	Organismal Systems	ko04712	0.007701	6	11
Base excision repair	17	Genetic Information Processing	ko03410	0.008901	3	14
Starch and sucrose metabolism	68	Metabolism	ko00500	0.010989	25	43
Glycosaminoglycan degradation	7	Metabolism	ko00531	0.013165	1	6
Diterpenoid biosynthesis	7	Metabolism	ko00904	0.013165	3	4
Zeatin biosynthesis	7	Metabolism	ko00908	0.013165	4	3
Linoleic acid metabolism	8	Metabolism	ko00591	0.013753	4	4
**200 mM NaCl-168 h vs. 200 mM NaCl + 10 mM KCl-168 h**						
Ribosome	438	Genetic Information Processing	ko03010	0.000000	352	86
Oxidative phosphorylation	156	Metabolism	ko00190	0.000000	107	49
Fatty acid elongation	21	Metabolism	ko00062	0.000000	17	4
Phenylpropanoid biosynthesis	36	Metabolism	ko00940	0.000000	24	12
Stilbenoid, diarylheptanoid and gingerol biosynthesis	8	Metabolism	ko00945	0.002351	5	3
Flavonoid biosynthesis	10	Metabolism	ko00941	0.002846	5	5
Glycolysis/Gluconeogenesis	73	Metabolism	ko00010	0.008388	51	22
Brassinosteroid biosynthesis	4	Metabolism	ko00905	0.026406	4	0
Glutathione metabolism	37	Metabolism	ko00480	0.033437	28	9
Limonene and pinene degradation	7	Metabolism	ko00903	0.050223	5	2
Cutin, suberine and wax biosynthesis	6	Metabolism	ko00073	0.053552	3	3
Biosynthesis of unsaturated fatty acids	20	Metabolism	ko01040	0.068133	18	2
Diterpenoid biosynthesis	4	Metabolism	ko00904	0.123174	1	3
Citrate cycle (TCA cycle)	52	Metabolism	ko00020	0.149175	33	19
Ascorbate and aldarate metabolism	18	Metabolism	ko00053	0.172390	11	7
Fatty acid metabolism	40	Metabolism	ko01212	0.177221	35	5
Sesquiterpenoid and triterpenoid biosynthesis	5	Metabolism	ko00909	0.185229	2	3
Plant hormone signal transduction	31	Environmental Information Processing	ko04075	0.189067	21	10
Arachidonic acid metabolism	7	Metabolism	ko00590	0.215454	5	2
Flavone and flavonol biosynthesis		Metabolism	ko00944	0.252624	0	1

**Table 2:** Antioxidant DEGs annotated to the KEGG pathways.

Pathway	Gene ID	Description	Log_2_ Fold-Change	
			200 mM NaCl-48 h vs. 200 mM NaCl + 10 mM KCl-48 h	200 mM NaCl-168 h vs. 200 mM NaCl + 10 mM KCl-168 h
SOD				
ko04146	*Unigene0033269*	SOD4 protein, partial	0.69	−0.06
	*Unigene0049419*	Superoxide dismutase [Mn]	−7.82	−9.54
	*Unigene0050462*	Superoxide dismutase	0.73	0.71
	*Unigene0082550*	Superoxide dismutase	0.25	−0.25
POD				
ko01100; ko01110; ko00940	*Unigene0009260*	Peroxidase 20	−1.66	−0.10
	*Unigene0013825*	Peroxidase	−1.87	0.99
	*Unigene0013827*	Peroxidase	−0.65	−0.09
	*Unigene0014843*	Peroxidase	3.20	1.25
	*Unigene0029752*	Peroxidase 17	−2.04	0.03
	*Unigene0049353*	Peroxidase 5	4.50	−1.35
	*Unigene0086491*	Peroxidase 52	−1.70	−0.59
	*Unigene0094375*	Peroxidase 31	1.10	−0.42
CAT				
ko01100; ko01110; ko01200; ko00630; ko04146; ko04016; ko00380	*Unigene0046159*	Catalase isozyme 1	0.77	0.25
	*Unigene0046160*	Catalase, partial	0.93	0.40
	*Unigene0087092*	Leaf catalase	−0.75	0.38
	*Unigene0103080*	Catalase isozyme 1	−5.74	−12.41
APX				
ko01100; ko00480	*Unigene0008032*	L-ascorbate peroxidase 3	0.01	0.21
	*Unigene0008033*	L-ascorbate peroxidase 3	−0.34	−0.43
	*Unigene0008513*	Peroxidase domain-containing	−1.50	0.47
	*Unigene0048033*	Cytosolic ascorbate peroxidase	0.62	0.13
	*Unigene0105664*	Thylakoid ascorbate Peroxidase precursor, partial	−0.35	−0.21
GPX				
ko01100; ko00480; ko00590	*Unigene0035407*	Glutathione peroxidase	−1.32	0.34
GST				
ko01100; ko00480	*Unigene0001041*	Glutathione S-transferase	−1.61	5.20
	*Unigene0004890*	Glutathione S-transferase T1-like	1.03	0.58
	*Unigene0007072*	Glutathione S-transferase U17-like	−1.13	−1.16
	*Unigene0012650*	Glutathione S-transferase Mu 1-like	−4.15	0.13
	*Unigene0015109*	Glutathione S-transferase U8-like	1.48	0.44
	*Unigene0020552*	Glutathione S-transferase	0.48	0.12
	*Unigene0041633*	Microsomal glutathione S-transferase 3-like	−0.24	−0.04
	*Unigene0048538*	Glutathione S-transferase U10-like	3.56	3.19
	*Unigene0056773*	Glutathione S-transferase	−0.04	−0.44
	*Unigene0064942*	Glutathione S-transferase L3	−0.47	0.33
	*Unigene0069058*	Glutathione-S-transferase	0.72	−0.06
	*Unigene0069060*	Glutathione S-transferase L3-like	−1.55	0.48
	*Unigene0081745*	Glutathione S-transferase U10-like	−1.08	−0.63
	*Unigene0082147*	Glutathione S-transferase F11-like	−3.80	0.49
	*Unigene0098941*	Glutathione S-transferase U9	−0.83	5.91
GR				
ko01100; ko00480	*Unigene0075696*	Glutathione reductase	−0.50	−0.10
	*Unigene0098587*	Glutathione reductase-like	−8.98	−10.52

**Table 3:** Information sheet for 21 species.

Family	Species	Description	Gene	Protein ID	CDS (bp)	ORF Length (aa)
Amaranthaceae	*Spinacia oleracea*	Glutathione S-transferase U10-like	*SolGST*	XP_021860277.1	693	230
Euphorbiaceae	*Ricinus communis*	Glutathione S-transferase U9	*RcoGST*	XP_002511574.1	696	231
Rhamnaceae	*Ziziphus jujuba var. spinosa*	Glutathione S-transferase U9-like	*ZjuGST*	XP_048330345.1	693	230
Malvaceae	*Herrania umbratica*	Glutathione S-transferase U9-like	*HumGST*	XP_021287145.1	693	230
Juglandaceae	*Carya illinoinensis*	Glutathione S-transferase U9	*CilGST*	XP_042947240.1	705	234
Amaranthaceae	*Chenopodium quinoa*	Glutathione S-transferase U10-like	*CquGST*	XP_021761389.1	693	230
Malvaceae	*Theobroma cacao*	Glutathione S-transferase tau 9	*TcaGST*	EOY21827.1	693	230
Rosaceae	*Pyrus*× *bretschneideri*	Glutathione S-transferase U9	*PbrGST*	XP_018505776.2	693	230
Juglandaceae	*Juglans regia*	Glutathione S-transferase U9-like	*JreGST*	XP_018848691.2	690	229
Rosaceae	*Prunus avium*	Glutathione S-transferase U10-like	*PavGST*	XP_021820694.1	696	231
Fagaceae	*Quercus suber*	Glutathione S-transferase U9-like	*QsuGST*	XP_023928011.1	699	232
Rosaceae	*Rosa chinensis*	Glutathione S-transferase U10	*RchGST*	XP_024173178.1	702	233
Fabaceae	*Prosopis alba*	Glutathione S-transferase U9-like	*PalGST*	XP_028763447.1	699	232
Rosaceae	*Prunus dulcis*	Glutathione S-transferase U9-like	*PduGST*	XP_034217038.1	693	230
Fagaceae	*Quercus lobata*	Glutathione S-transferase U9	*QloGST*	XP_030937552.1	699	232
Rutaceae	*Citrus sinensis*	Glutathione S-transferase U9-like	*CsiGST*	XP_015385201.1	693	230
Rosaceae	*Prunus persica*	Glutathione S-transferase U10	*PpeGST*	XP_007208953.1	699	232
Rubiaceae	*Coffea arabica*	Glutathione S-transferase U9-like	*CarGST*	XP_027124538.1	705	234
Myricaceae	*Morella rubra*	Glutathione S-transferase U9	*MruGST*	KAB1199317.1	696	231
Cannabaceae	*Cannabis sativa*	Glutathione S-transferase U9	*CsaGST*	XP_030492967.1	693	230
Vitaceae	*Vitis riparia*	glutathione S-transferase U9-like	*VriGST*	XP_034673741.1	696	231

## Data Availability

Not applicable.

## Supplementary Materials

The following supporting information can be downloaded at: https://www.mdpi.com/article/10.3390/genes13091507/s1, Table S1: Sequence of specific primers, Table S2: log_2_ fold-change of 10 DEGs at 48 h and 168 h after exogenous potassium (K^+^) application under NaCl stress, Figure S1: Expression level of *Unigene0048538* in GST activity, Figure S2: Verification of DEGs by qRT-PCR.
