# Supplementary material for: Effects of Exogenous Potassium (K+) Application on the Antioxidant Enzymes Activities in Leaves of Tamarix ramosissima under NaCl Stress
Source: Genes (Basel). 2022 Aug 23;13(9):1507. doi: 10.3390/genes13091507 (PMC9498862; doi:10.3390/genes13091507)
Supplement: Supplementary file 1 [file genes-13-01507-s001.zip › genes-1848210-supplementary.pdf]

Supplementary Materials

Table S1. Sequences of specific primers.

Primer name	Primer sequence (5′–3′)
Unigene0104732	F: TCCCCTCACCAGTGACCACCA<br>R: TGAGGAAGCGGGAGAACGCCA
Unigene0083695	F: AGGAAGCGTCCATGGGGCGA<br>R: AACTCGTCCACCGCAAGCCG
Unigene0069097	F: AGCAGCCGATTGTCTCCTTGGGA<br>R: GCACTGCTCCTTTCTCCCTCTGC
Unigene0024962	F: ACCATGTCGGCCCGCTTGAC<br>R: TCCGCTGCAGTGGCCCTAGT
Unigene0007135	F: AGGAAGGCGGTGAGGGTGCT<br>R: GCAGCACCGGGAGTCGTAGC
Unigene0088781	F: GGTGGTGGCGGCGGTGATAC<br>R: TGCTGCAACTGCCGCTCCTC
Unigene0028215	F: TCATGAACGGCGCCAGCCAA<br>R: TGGAGGACCGCACCGCCATA
Unigene0082586	F: ACCGACGCAGGGAAGGTGGT<br>R: GCCCGCTCACAGTCGAAGCC
Unigene0003066	F: CGACGGGAGGCGTAGGGGAA<br>R: GCAGCCTGCGAACACATCCCA
Unigene0090596	F: TCCCGCAGTACCTGCTCACGA<br>R: TGGAGACCCCGACGAGGTGG
Actin	F: TCGTAGCAGAGCATCGGAGAA<br>R: TGACCCATGCCAACCATAACA

F means forward primer, R means reversed primer.

Table S2. Expression changes of 10 DEGs after exogenous potassium (K^+) application under NaCl stress.

Gene ID	Description	$Log_2FoldChange$	
		NaCl-48h vs. NaCl + KCl-48h	NaCl-168h vs. NaCl + KCl-168h
Unigene0104732	Transcription factor bHLH48-like	−1.05	0.25
Unigene0083695	bZIP transcription factor 44-like	−0.11	1.33
Unigene0069097	vacuolar membrane Na^+/H^+ antiporter	−0.30	−0.11
Unigene0024962	WRKY transcription factor 1	0.27	−0.01
Unigene0007135	WRKY33-1	0.51	0.57
Unigene0088781	Transcription factor MYB4-like	0.62	−0.45
Unigene0028215	Predicted: transcription factor bHLH112 isoform X1	1.64	1.07
Unigene0082586	SAP30-binding protein isoform X2	0.07	−0.02
Unigene0003066	Transcription factor MYB44-like	0.74	−0.22
Unigene0090596	Sodium transporter HKT1	1.36	0.36

NaCl means 200 mM NaCl; NaCl + KCl means 200 mM NaCl + 10 mM KCl.

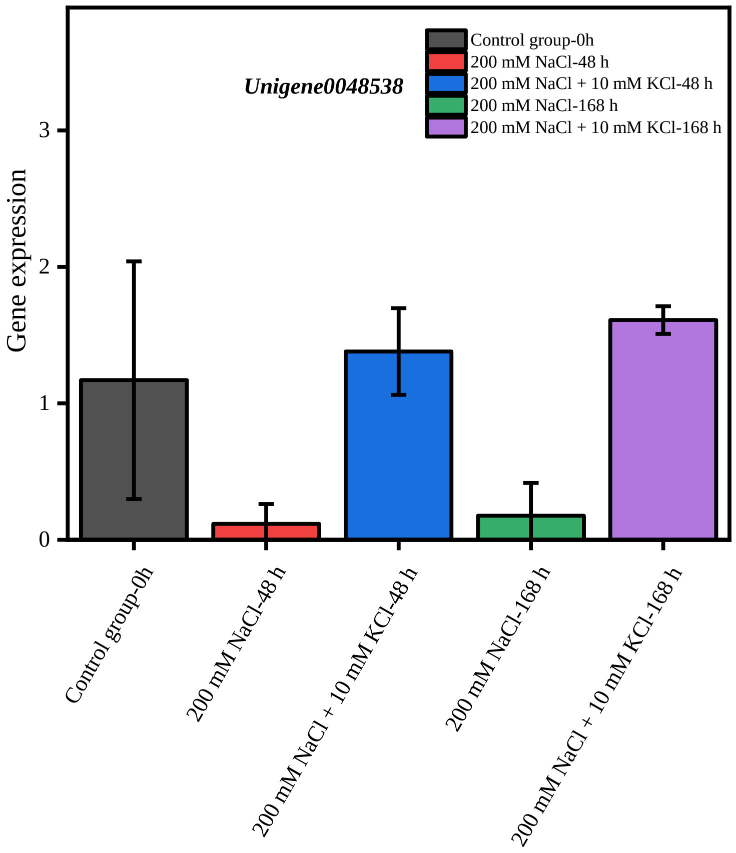

Figure S1. Expression level of *Unigene0048538* in GST activity. Changes in expression levels of *Unigene0048538* in GST activity under different treatments.

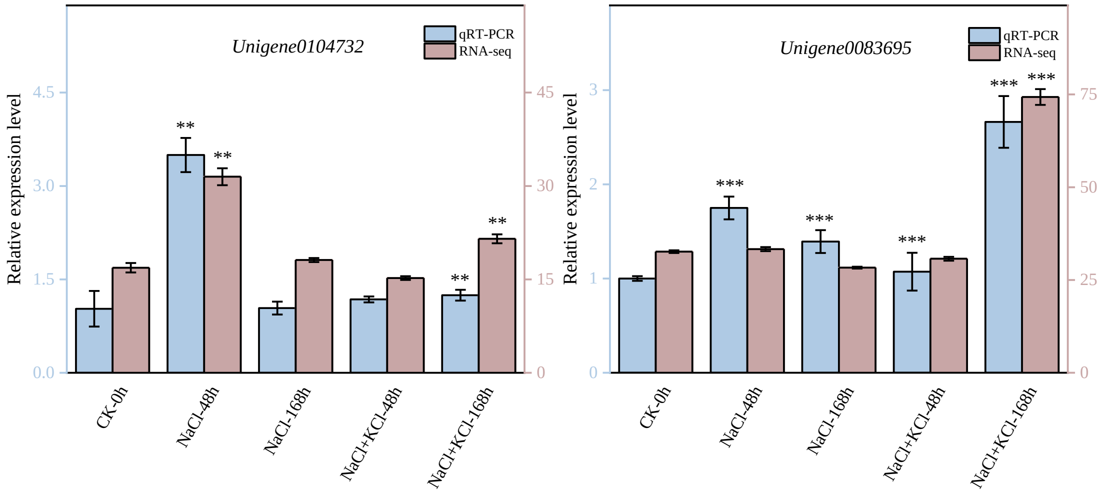

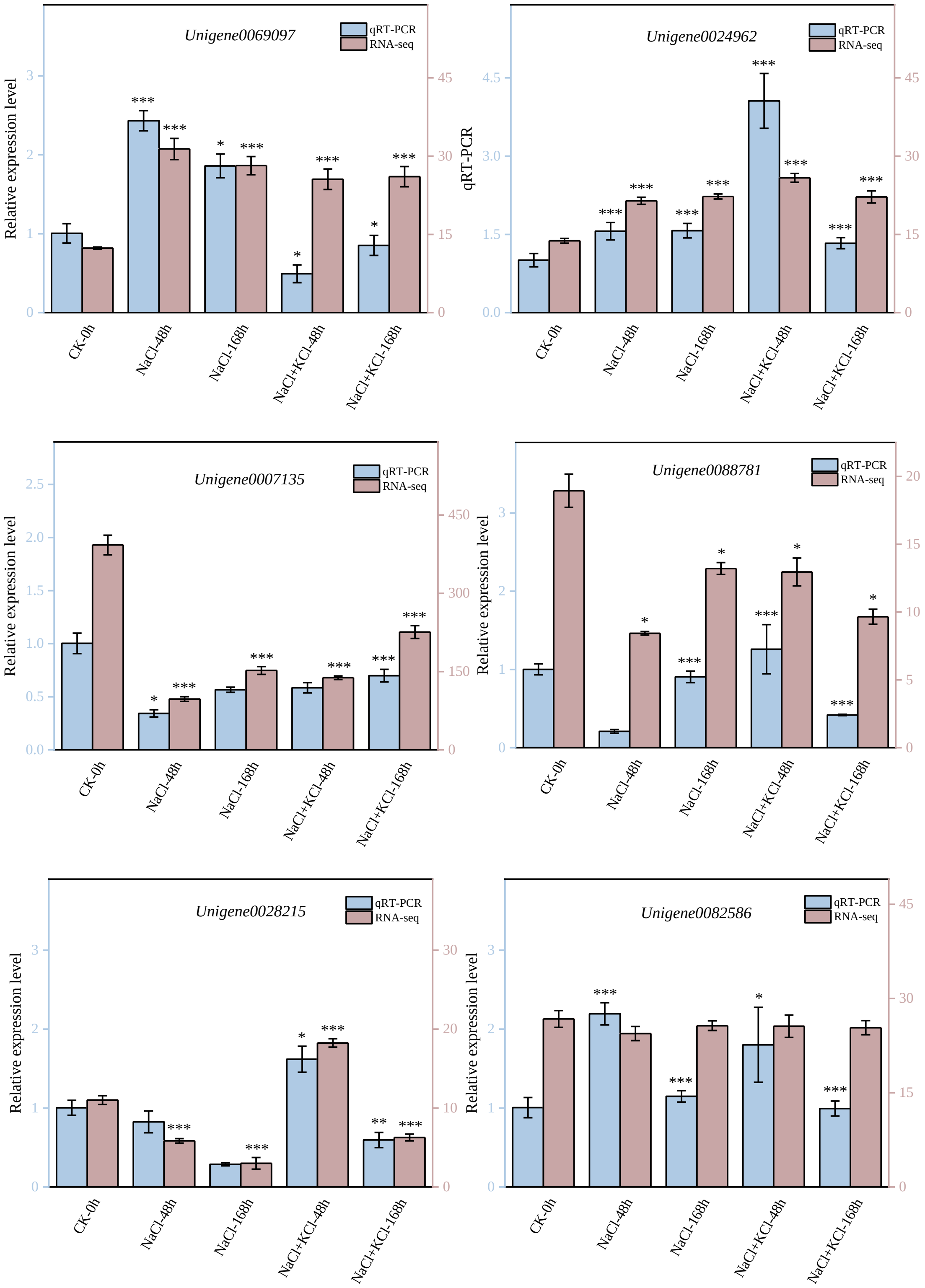
Unigene0069097
Unigene0024962
Unigene0007135
Unigene0088781
Unigene0028215
Unigene0082586
qRT-PCR
RNA-seq
Relative expression level
qRT-PCR
CK-0h
NaCl-48h
NaCl-168h
NaCl+KCl-48h
NaCl+KCl-168h

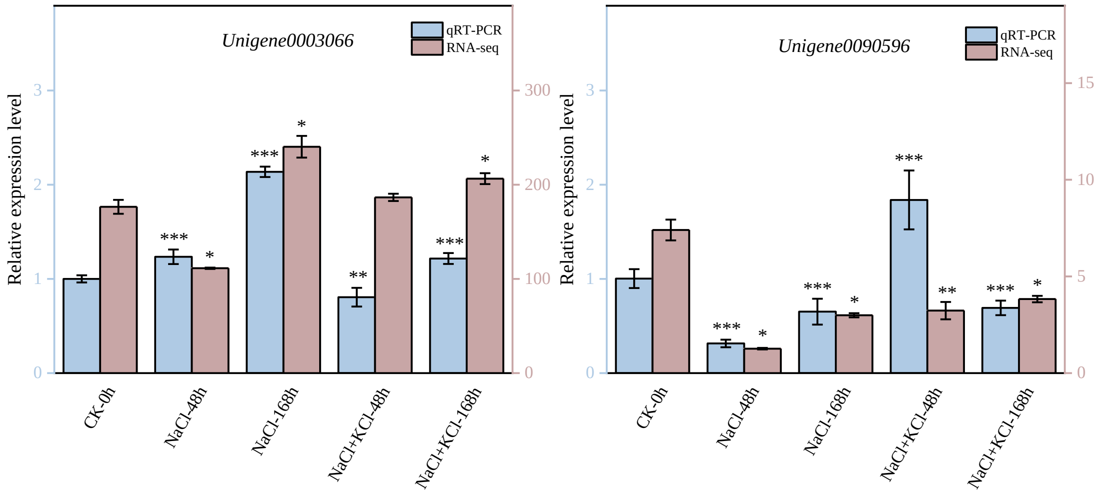

Figure S2. Validation of DEGs by qRT-PCR. 10 DEGs were randomly selected for qRT-PCR validation, and the error bars were obtained from multiple replicates of qRT-PCR.

Note: qRT-PCR : gene expression levels are shown on the left; RNA-seq: gene expression levels are shown on the right; $p \geq 0.05$ is not marked, $0.01 < p < 0.05$ is marked as *; $0.001 < p < 0.01$ is marked as **; $p \leq 0.001$ is marked as ***.
